# Investigating Mental Health Service User Opinions on Clinical Data Sharing: Qualitative Focus Group Study

**DOI:** 10.2196/30596

**Published:** 2021-09-03

**Authors:** Abimbola Adanijo, Caoimhe McWilliams, Til Wykes, Sagar Jilka

**Affiliations:** 1 Department of Psychology Institute of Psychiatry, Psychology and Neuroscience King's College London London United Kingdom; 2 South London and Maudsley NHS Foundation Trust London United Kingdom; 3 Division of Mental Health & Wellbeing Warwick Medical School University of Warwick Coventry United Kingdom

**Keywords:** clinical data, data sharing, mental health data, service users, focus groups, mental health, digital health, health records

## Abstract

**Background:**

Sharing patient data can help drive scientific advances and improve patient care, but service users are concerned about how their data are used. When the National Health Service proposes to *scrape* general practitioner records, it is very important that we understand these concerns in some depth.

**Objective:**

This study aims to investigate views of mental health service users on acceptable data sharing to provide clear recommendations for future data sharing systems.

**Methods:**

A total of 4 focus groups with 4 member-checking groups were conducted via the internet between October 2020 and March 2021, with a total of 22 service users in the United Kingdom. Thematic analysis was used to identify the themes.

**Results:**

Six main themes, with several subthemes were identified, such as the *purpose* of data sharing—for profit, public good, and continuation of care; *discrimination* through the misattribution of physical symptoms to mental health conditions (ie, diagnostic overshadowing) alongside the discrimination of individuals or groups within society (ie, institutional discrimination); *safeguarding* data by preserving anonymity and confidentiality, strengthening security measures, and holding organizations accountable; data *accuracy* and *informed consent*—increasing transparency about data use and choice; and incorporating *service user involvement* in system governance to provide insight and increase security.

**Conclusions:**

This study extends the limited research on the views and concerns of mental health service users regarding acceptable data sharing. If adopted, the recommendations should improve the confidence of service users in sharing their data. The five recommendations include screening to ensure that data sharing benefits the public, providing service users with information about how their data are shared and what for, highlighting the existing safeguarding procedures, incorporating service user involvement, and developing tailored training for health care professionals to address issues of diagnostic overshadowing and inaccurate health records. Adopting such systems would aid in data sharing for legitimate interests that will benefit patients and the National Health Service.

## Introduction

### Background

Patient-level clinical data are increasingly recognized as a valuable resource that can help drive scientific advances and innovations to improve patient care [1]. Global initiatives actively promote and enable data sharing, and in research, most funders mandate researchers to plan for sharing their data [2-4]. However, to facilitate responsible data sharing, we need to develop systems that work for all stakeholders, and mental health service users need to be central to these developments. We already know that people with depression, epilepsy, and multiple sclerosis all have concerns about how their data would and should be used in health services [5-9]; however, these have not been explored in depth, and we do not know what service users consider to be acceptable limits for sharing their data. As the National Health Service (NHS) currently plans to scrape data from general practitioner practices [10], any concerns are likely to affect the legitimacy of such actions and potentially undermine the trust of mental health service users.

The Academy of Medical Sciences published a report on harnessing NHS data for future health benefits together with a dialogue report of conversations with NHS patients and the public on data use [11,12]. One challenge that surfaced is the continued protection of privacy, a particular concern for mental health service users [13]. The report also highlighted the balance between maintaining confidence in safeguarding data and enabling appropriate access to data-driven technologies. Many principles of data sharing are important for patients and the public, but there is considerable sensitivity among those with mental health problems because of stigma and discrimination [14]. There were few such individuals in the data dialogue study [11], but even when these individuals were asked or when others commented on mental health case studies, more skepticism was exhibited.

A previous qualitative study that investigated the views of service users about sharing administrative data [15] found that participants were largely comfortable sharing health records, including sensitive mental health data, with organizations that they trust. Trust was contingent on high transparency (ie, clarity on how this information would be shared and used), service user autonomy (ie, the ability of service users to have a say in the sharing of their data), and adequate security (ie, guarantees that the data shared would be adequately protected). However, this was a very small study (N=8); therefore, the themes reported are not generally applicable to the mental health population because of the geographic, age, and size limitations of their sample [15].

### Objectives

There are various accepted data sharing and use models that exist domestically and internationally, but the technological landscape for data to be shared, integrated, and analyzed is constantly evolving. Therefore, there is a clear need for the views of service users to be represented in the development and governance of future data storage and use systems. This study investigates service user views to demarcate the boundaries of acceptable data use and sharing and provide clear recommendations for future systems.

## Methods

### Design

This is a qualitative study with focus groups conducted virtually between October 15, 2020, and March 15,2021, using a videoconferencing software (Microsoft Teams) because of COVID-19. Focus groups followed a topic guide that contained open questions on sharing clinical data and their views on how systems should be developed to ensure that future data sharing initiatives are ethical and efficient. Each focus group was also followed with a member-checking focus group [16], to view the initial data analysis.

### Recruitment

Participants were eligible if they were aged at least 18 years and had experience using mental health services. Participants needed internet or phone access and were excluded if they were unable to provide informed consent. They were recruited through purposive sampling via existing patient involvement groups and a research register (Consent for Contact) held by the South London and Maudsley NHS Foundation Trust (SLaM).

### Focus Groups

The *topic guide* was based on previous data sharing research [15] and was expanded to include other issues that became apparent with changes in technology. The guide explored what participants thought about different data sharing models; how participants felt about their clinical data being shared with other hospitals, universities, government organizations, and companies; specific concerns about data sharing; how data can be shared (eg, raw data or aggregated summary data); their boundaries for what information can be shared; and how their trust can be earned about how their data are shared.

Participants were provided with a summary paper outlining current data sharing systems to provide background to the topic (Multimedia Appendix 1). This was referred to and summarized at the start of each focus group to ensure that participants had the same baseline prerequisite knowledge. In brief, the summary sheet includes the following information:

1. *NHS Digital’s Hospital Episode Statistics* data, which contain more than 1 billion records of patient service attendances across hospitals commissioned by England’s NHS Clinical Commissioning Groups [17].
2. The *Clinical Record Interactive Search* (CRIS) system, which provides authorized researchers with regulated access to anonymized patient-level data that are extracted from the SLaM electronic clinical records system. CRIS was developed with service user input on data protection issues, and applications were reviewed by a CRIS oversight committee, chaired by a service user. All data remained within the NHS firewall [18,19].

We also discussed the following potential adaptations to the CRIS data sharing system:

1. Extending CRIS by amalgamating data with other NHS trusts to provide a larger database so we can ask more questions. The data would be anonymous, and access will be governed by a committee as in the SLaM CRIS system. In this model, the data are outside the NHS firewall.
2. Developing separate CRIS databases within each individual NHS trust. These data would also be anonymous and follow similar rules as that of the SLaM CRIS system; however, the data would still be accessible within the NHS firewall of each NHS trust.

Each focus group lasted up to 2 hours, and each member-checking group lasted up to 1 hour; they were all digitally recorded using Microsoft Teams, and the recordings were transcribed manually.

### Procedure

The study was approved by the East of the Scotland Research Ethics Committee (ref. 20/ES/0004). Participants provided written informed consent and their self-reported clinical and demographic characteristics before each of the 2 focus groups. The first focus group discussed acceptable data sharing, which was then analyzed to identify relevant themes and construct an initial thematic map. The second *member-checking* group considered the thematic map and provided feedback as a check that service users agreed with the emerging themes. Participants were reimbursed for their participation, and researchers supported the participants’ well-being and welfare throughout the study.

### Data Analysis

Data collection continued until we reached data saturation, which was established by a review of the summary findings from each focus group (Multimedia Appendix 2) [20]. We used thematic analysis, following the six stages prescribed by Braun and Clarke [21]:

- Stage 1: The focus group recordings were manually transcribed to facilitate data immersion, and the recordings were listened to multiple times to ensure accurate transcription.
- Stage 2: Two service user researchers independently coded the data using an inductive coding approach.
- Stage 3: Once the initial codes were developed, potential themes were identified by combining codes to produce thematic maps.
- Stage 4: These themes were reviewed to establish relevance to the research question and ensure that they were coherent and distinctive. Any themes that were not supported with sufficient data or deemed too discrete were discarded.
- Stage 5: Relevant themes were clearly defined and named and then combined into a final thematic map.
- Stage 6: Relevant extracts from the transcripts were chosen to illustrate each theme. NVivo 12 software (QSR International) was used to manage and code the data.

## Results

### Sample Characteristics

A total of 22 people aged between 21 and 74 years (mean 45.04, SD 16.29 years) participated. The majority were women (15/22, 68%), and we had a diverse sample with only 55% (12/22) White British. The remaining samples were White European, Asian or Asian British, Black or Black British, or mixed White and Black Caribbean. Of the total participants, 91% (20/22) were diagnosed with a mental health condition (eg, depression or anxiety) and 64% (14/22) were educated to the degree level. Approximately 68% (15/22) of the participants resided in London (Southeast England), and the remaining participants lived in different regions of England (eg, West Midlands, East Midlands, and Southwest England).

### Themes

#### Overview

**Figure 1:**
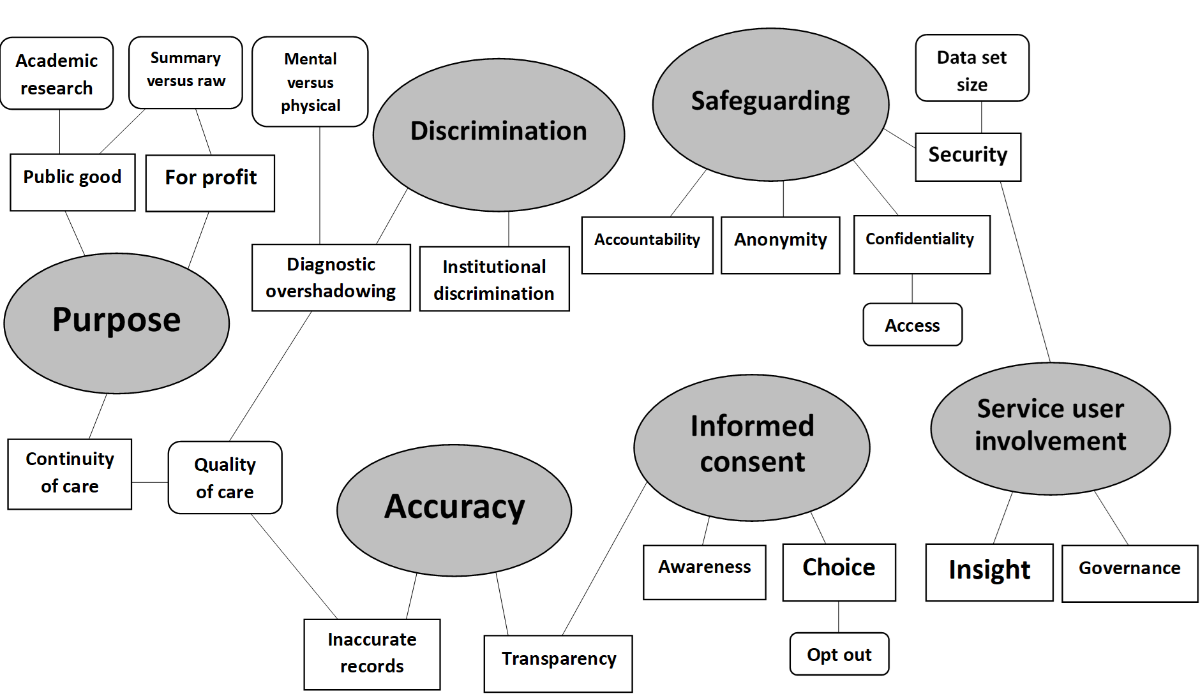
Thematic map showing six main themes and 22 subthemes.

Each theme and subtheme included in the final thematic map was discussed in at least two of the 4 focus groups (Multimedia Appendix 2). Figure 1 illustrates a summary of the six main themes and 22 subthemes. Service users found some existing models acceptable (eg, SLaM’s CRIS system) [18] because service users were involved in their development. Despite accepting this model, there were conflicting opinions about pooling data across different NHS trusts versus each NHS trust establishing their own CRIS-style data sharing system. Service users were concerned about the impact on security and diagnostic overshadowing (ie, where health care professionals appeared to dismiss their physical illnesses when made aware of their mental health diagnosis [22]), if CRIS was extended across NHS trusts, but they recognized the benefit of continuity of care. We subsequently describe each theme in detail.

#### Purpose

Service users expressed that they felt uncomfortable with their data being shared with commercial companies that used this information for their own financial gain, that is, *for profit*:

The concept of selling people’s data to a company for profit is completely unacceptable.

Participant 21

However, service users were comfortable sharing data that contributed to *the public good*, such as *academic research*:

I feel quite comfortable about researchers and letting universities access it because I feel like the intention is to see health trends and to do good.

Participant 3

Although service users generally found data sharing within the NHS acceptable and in line with the *public good* narrative, they did acknowledge that there was some skepticism because of the increasing privatization. However, it could be argued that the skepticism was because of NHS privatization in general, rather than solely in the context of data sharing:

Because of all this privatization of the NHS, which is very concerning for most of us, I signed a petition last week, because 49 GP surgeries have been sold to an American health insurance company. So that to me is very concerning.

Participant 21

The extent to which service users were comfortable with organizations having access to *summary versus raw data* (or aggregated vs patient-level data) was also dependent on the purpose. Generally, service users felt it was more appropriate for summary data only to be provided when data were shared for profit, but when data were shared to benefit the public, service users voiced that organizations should have access to raw data to maximize use:

I would prefer summary [data] because companies like Google - the more information they have, they exploit it. So, I would say a summary can still help them to do research if they need to.

Participant 4

It was also felt that sharing data to facilitate *continuity of care* for patients in health care settings was acceptable and could improve the *quality of care* service users received:

I couldn’t agree more with what you just said for continuity purposes. For example, ...you end up in another city, then you would hope they have access to the medical information - know what drugs you are on and so on so they can treat you quickly and correctly.

Participant 2

...data sharing is quite important in terms of improving services or reducing the amount of time you have to keep explaining to different people about who you are, what you are, what you’re doing.

Participant 9

Service users suggested that extending CRIS and having data from different NHS trusts collated in one place could be beneficial for continuity of care in health care settings:

...if you’re accessing different hospitals and then there’s not an exchange of information, sometimes you can think there’s no continuity.

Participant 3

#### Discrimination

Within health care settings, service users recounted experiencing *diagnostic overshadowing*:

I have been basically stigmatized and dismissed when attending A&E for physical health concerns because they saw my mental health diagnosis. So, often in that situation, I wish it worked separately.

Participant 2

Service users believed that data sharing within health care settings could also be detrimental to the *quality of care* when discriminatory beliefs or actions were present. As a result, service users were more comfortable with their *physical health data being shared than their mental health data*:

People with mental health conditions generally get poorer physical health care as they are often not believed...so many of us with mental health histories would prefer clinicians who are treating our physical ailments not to know about our mental health.

Participant 13

...there’s a discrepancy between physical health and mental health for me personally...I don’t really mind if somebody knew that I’d broken my leg, for example, but actually I would mind if somebody knew that I went to see a doctor about my mental health.

Participant 3

Service users also voiced concerns that were centered on discrimination toward individuals or groups in society, that is, *institutional discrimination*. It was felt that data sharing could lead to people being scapegoated or restricted from doing or receiving certain things, given the historical discrimination (eg, with Romani and gay communities):

Well it [the data] could be used, I suppose to stigmatise people or to prevent people from having, for example, the benefits that they are entitled to...

Participant 1

Research was done into lots of communities, not just the Jewish community; Romani communities and lots of other communities in which the purpose wasn’t for the benefit of science. It was to discriminate...so lots of Romani people today feel very reluctant about giving information because they’re scared about where it may go.

Participant 7

#### Accuracy

Service users reported having *inaccurate clinical records* and the negative impact that this had on their *quality of care*:

I do worry of false information that is there, that might have been entered years ago and nobody ever bothered to check...because it’s simply inaccurate.

Participant 2

To address this, service users wanted more *transparency* from health care professionals regarding the content of their clinical notes. They wanted the opportunity to view their records to check that the information was accurate and if it was found to be inaccurate, to either have this amended or their disagreement noted in their records:

I mean for me it’s even more important that I had chance to regularly look at that information and check that it’s the correct information.

Participant 1

#### Informed Consent

It was felt that there needed to be more *transparency* from organizations about what the data were being used for:

I think that people need to feel that their data is being used correctly. So, I think that they have to be more open and say we will use your data for this...

Participant 19

They also discussed the need to increase *awareness* among service users about the data sharing process, as not all service users were aware that their data could be shared this way:

I think it’s really important that everybody, like every member of the public, knows that their data can be accessed because I didn’t personally know that a researcher could access it in that way until I was doing this.

Participant 3

Furthermore, service users wanted to have a *choice* in the sharing of their data after being appropriately informed, including the option to *opt out* of data sharing if they were not comfortable:

Just give the choice to the patient to decide which information to give out.

Participant 10

#### Safeguarding

This theme considers the importance of storing and sharing data securely. Service users discussed the need to maintain *confidentiality*, with more consideration of who has *access* to sensitive data and preserving service user *anonymity* when sharing data:

...there might be a few things that you really need to share with the GP or a professional to see why you’re feeling the way you’re feeling or what’s causing you the mental health issues or physical health issues, but you don’t really want anyone else to read certain parts.

Participant 9

I think as long as it’s anonymized, that’s fine.

Participant 19

Service users were concerned about the *security* of data systems and the increased risk to security with a *larger data set*. For this reason, there was some hesitancy about expanding the CRIS system to pool all the data from different NHS trusts into one place:

I think in general it’s best not to put all your eggs in one basket [with the third CRIS model] and there is more of a security risk from pulling everything together. It also makes it much more likely to be the target of an attack.

Participant 13

...the bigger it gets, the more I trust that the data wouldn’t be secure.

Participant 17

It was felt that organizations needed to be held *accountable* for adhering to *safeguarding* protocols; if these protocols were breached, organizations should be penalized, and service users should be appropriately compensated:

...very much falls on the organisations to be accountable for making sure that they are adhering to data protection, GDPR, data storage, data sensitivity.

Participant 9

...it would have to be a significant compensation measure for a person who potentially could have their lives ruined by a data leak and if that was in place, I’d probably feel a lot more trusting of it [data sharing], and more free with the idea of that going ahead.

Participant 18

#### Service User Involvement

There was a particular emphasis on service users being involved in the *governance* of data sharing systems, as is currently the case within the CRIS data sharing model. Service users felt that there was a need for a service user perspective to provide invaluable *insight* and make people feel more *secure* in sharing their data:

I would want service users to be involved in some of that governance.

Participant 1

When you talk about CRIS and how the data are protected, I think it’s wonderful that you’re using it chaired by a service user and service user input is very valid throughout the protection system.

Participant 14

...in terms of an additional security measure, you’ve got a group of trusted people [service users] who decide on what to do with the request...

Participant 18

## Discussion

### Principal Findings

Trust has again been identified as a key component to effective engagement in research and health care settings [23]. Service users were hesitant about sharing data with commercial companies as they were mistrustful of their intentions, which were largely believed to be unethical and purely for profit. Currently, there is a limited sharing of data to commercial companies directly [24], although some commercial companies do register potential customers as pharmaceutical companies and medical device services.

Service users felt more comfortable sharing sensitive data within the NHS and with academic institutions as they had more confidence that the information would be used for public benefit, which mirrors the existing literature [11,15]. However, they also mentioned that increasing NHS privatization was beginning to affect their trust [25,26].

Service user trust is contingent on high transparency and service user autonomy [15], which was mirrored in our data, especially choice in the sharing of their data after learning how it is to be used. The current plan for data scraping and sharing by the NHS is being rolled out quietly and with little publicity. Although this is not suspicious, the lack of transparency is likely to undermine the confidence and reduce the overall worth of the data if many people decide to opt out. We recommend that future systems incorporate comprehensive screening processes to ensure that data sharing between organizations benefits the public and provides service users with adequate information about how their data are shared, and what for, to enable them to make an informed choice.

This concept of transparency can also be applied to health care settings. Service users wanted health care professionals to be more transparent with them about the content of their clinical notes, to resolve concerns about inaccurate health care records. Solutions proposed by service users in our study included ensuring that information is double-checked with patients before entering it and allowing service users to frequently view their health care records and dispute inaccuracies. Transparent medical records enhance trust, improve relationships with professionals, and increase understanding of health information [27,28]; however, there are concerns about service users reacting negatively to their content because of misinterpretation or misunderstanding, which could result in mistrust of health care professionals [28]. For transparency to be effective, the information within health care records must be communicated effectively and understood by the service user. We recommend increasing the transparency of clinical records and developing bespoke training for health care professionals in clinical data input and the communication of clinical information.

Discrimination within health care settings can have a negative effect on people’s trust in the health care system [29]. Existing studies indicate heightened skepticism about data sharing among individuals with mental health problems [11]. This is because of the stigma and discrimination experienced by service users within health care settings, resulting in poorer quality health care for people with mental health difficulties [14,30]. The issue of diagnostic overshadowing is not a new problem for service users, and we found that our service users’ experiences were not dissimilar to the findings in existing literature [31-34]. Improving clinical health care skills and knowledge can increase competence, reduce symptom misattribution, and encourage staff to reflect on their attitudes to prevent diagnostic overshadowing [30]. As a result, we recommend tailored training for nonmental health professionals to develop these skills.

Our results support the existing literature that highlights security and protection of privacy as prominent service user concerns, with trust contingent on adequate security [11-13,15]. Service users expressed the need to establish effective safeguarding measures and hold organizations accountable for any breach. Perceptions of security and privacy are positively correlated with trust, and greater perceptions of trust increase the likelihood of information sharing [35]. We recommend providing service users with clear information on existing General Data Protection Regulation procedures that are in place to protect patient data and hold organizations accountable for any data breach. Using trusted research environments may also be another solution to address security concerns related to sharing patient-level data [36].

Service user involvement was identified as an important factor to consider when developing future data sharing systems because it made them feel more confident. Service users have reported more open attitudes and improved trust in research as a result of involvement [37]. We recommend involving service users in the governance of future data systems to ensure that they are prioritized.

In terms of future research, there is a need to understand the acceptable levels of pseudonymization of data, as it is not understood how much data would breach the high public expectations of privacy. As more data are likely to be collated, for example, in the general practitioner records data scraping [10], information from reduced post codes, criminal records, and other data will affect the chance of patient identification. Although this may be acceptable for high levels of patient benefit, this should not go unchallenged or agreed upon by the public, especially those who use mental health services.

### Strengths and Limitations

The web-based nature of the study could have excluded some participants who did not have access to the required technology or who lacked digital competency [38,39]. Most of the participants were women. Although the current literature does not suggest differences in views, it is possible that the views of men and those from more diverse backgrounds might have weighted them differently. However, through our remote recruitment and data collection, it was more convenient and flexible for service users to participate and allowed participation from service users in geographically dispersed locations [40]. Our sample also fulfilled qualitative sample size criteria (N=22) [20,41,42] and provided views across a wider age range.

### Implications and Conclusions

This study extends the limited research available on service user views and concerns regarding acceptable data sharing and provides a foundation for further research. We make five main recommendations to build service user trust in data sharing: (1) comprehensive screening processes, (2) developing tailored training for health care professionals to tackle diagnostic overshadowing and inaccurate health records, (3) providing service users with adequate information, (4) highlighting existing safeguarding procedures, and (5) incorporating service user involvement.

Although the qualitative nature of this study allowed us to obtain rich and detailed data, we found it challenging to clearly determine service user preferences for specific data sharing models. Future research should focus on conducting discrete choice experiments to quantify service user preferences and conclusively determine what models service users deem more acceptable for clinical data sharing in the United Kingdom.

## Appendix

Multimedia Appendix 1 Summary sheet.

Multimedia Appendix 2 Saturation grid of themes and subthemes represented in focus groups.

## Abbreviations

CRIS: Clinical Record Interactive Search
NHS: National Health Service
SLaM: South London and Maudsley National Health Service Foundation Trust

## References

[1] Kochhar S, Knoppers B, Gamble C, Chant A, Koplan J, Humphreys GS (2019). Clinical trial data sharing: here's the challenge. BMJ Open.

[2] Institute of Medicine (IOM) (2015). Sharing Clinical Trial Data: Maximizing Benefits, Minimizing Risk.

[3] Kiley R, Peatfield T, Hansen J, Reddington F (2017). Data sharing from clinical trials - a research funder's perspective. N Engl J Med.

[4] Taichman D, Backus J, Baethge C, Bauchner H, de Leeuw PW, Drazen JM, Fletcher J, Frizelle FA, Groves T, Haileamlak A, James A, Laine C, Peiperl L, Pinborg A, Sahni P, Wu S (2016). Sharing clinical trial data: a proposal from the International Committee of Medical Journal Editors. Rev Med Chil.

[5] Bruno E, Simblett S, Lang A, Biondi A, Odoi C, Schulze-Bonhage A, Wykes T, Richardson MP, RADAR-CNS Consortium (2018). Wearable technology in epilepsy: The views of patients, caregivers, and healthcare professionals. Epilepsy Behav.

[6] Simblett S, Greer B, Matcham F, Curtis H, Polhemus A, Ferrão J, Gamble P, Wykes T (2018). Barriers to and facilitators of engagement with remote measurement technology for managing health: systematic review and content analysis of findings. J Med Internet Res.

[7] Simblett SK, Bruno E, Siddi S, Matcham F, Giuliano L, López JH, Biondi A, Curtis H, Ferrão J, Polhemus A, Zappia M, Callen A, Gamble P, Wykes T, RADAR-CNS Consortium (2019). Patient perspectives on the acceptability of mHealth technology for remote measurement and management of epilepsy: A qualitative analysis. Epilepsy Behav.

[8] Simblett SK, Evans J, Greer B, Curtis H, Matcham F, Radaelli M, Mulero P, Arévalo MJ, Polhemus A, Ferrao J, Gamble P, Comi G, Wykes T, RADAR-CNS consortium (2019). Engaging across dimensions of diversity: A cross-national perspective on mHealth tools for managing relapsing remitting and progressive multiple sclerosis. Mult Scler Relat Disord.

[9] Simblett S, Matcham F, Siddi S, Bulgari V, di San Pietro CB, López JH, Ferrão J, Polhemus A, Haro JM, de Girolamo G, Gamble P, Eriksson H, Hotopf M, Wykes T, RADAR-CNS Consortium (2019). Barriers to and facilitators of engagement with mhealth technology for remote measurement and management of depression: qualitative analysis. JMIR Mhealth Uhealth.

[10] (2021). General Practice Data for Planning and Research (GPDPR). NHS Digital.

[11] (2018). Our data-driven future in healthcare: People and partnerships at the heart of health related technologies. Academy of Medical Sciences.

[12] Castell S, Robinson L, Ashford H (2018). Future data-driven technologies and the implications for use of patient data: Dialogue with public, patients and healthcare professionals. Academy of Medical Sciences.

[13] Robotham D, Satkunanathan S, Doughty L, Wykes T (2016). Do we still have a digital divide in mental health? A five-year survey follow-up. J Med Internet Res.

[14] Knaak S, Mantler E, Szeto A (2017). Mental illness-related stigma in healthcare: Barriers to access and care and evidence-based solutions. Healthc Manage Forum.

[15] Satinsky EN, Driessens C, Crepaz-Keay D, Kousoulis A (2018). Mental health service users' perceptions of data sharing and data protection: a qualitative report. J Innov Health Inform.

[16] Birt L, Scott S, Cavers D, Campbell C, Walter F (2016). Member Checking: A tool to enhance trustworthiness or merely a nod to validation?. Qual Health Res.

[17] (2016). Hospital Episode Statistics. NHS Digital.

[18] Perera G, Broadbent M, Callard F, Chang C-K, Downs J, Dutta R, Fernandes A, Hayes RD, Henderson M, Jackson R, Jewell A, Kadra G, Little R, Pritchard M, Shetty H, Tulloch A, Stewart R (2016). Cohort profile of the South London and Maudsley NHS Foundation Trust Biomedical Research Centre (SLaM BRC) Case Register: current status and recent enhancement of an Electronic Mental Health Record-derived data resource. BMJ Open.

[19] (2016). CRIS blog: Eight years on. NIHR Maudsley BRC.

[20] Guest G, Namey E, McKenna K (2016). How many focus groups are enough? Building an evidence base for nonprobability sample sizes. Field Methods.

[21] Braun V, Clarke V (2006). Using thematic analysis in psychology. Qual Res Psychol.

[22] Jones S, Howard L, Thornicroft G (2008). 'Diagnostic overshadowing': worse physical health care for people with mental illness. Acta Psychiatr Scand.

[23] Wilkins C (2018). Effective engagement requires trust and being trustworthy. Med Care.

[24] (2021). Data Release Register. NHS Digital.

[25] Triggle N (2015). NHS privatisation: Why the fuss?. BBC News.

[26] Iacobucci G (2019). Is the NHS being privatised?. Br Med J.

[27] Esch T, Mejilla R, Anselmo M, Podtschaske B, Delbanco T, Walker J (2016). Engaging patients through open notes: an evaluation using mixed methods. BMJ Open.

[28] Erlingsdóttir G, Petersson L, Jonnergård K (2019). A theoretical twist on the transparency of open notes: qualitative analysis of health care professionals' free-text answers. J Med Internet Res.

[29] Rivenbark JG, Ichou M (2020). Discrimination in healthcare as a barrier to care: experiences of socially disadvantaged populations in France from a nationally representative survey. BMC Public Health.

[30] Nash M (2013). Diagnostic overshadowing: a potential barrier to physical health care for mental health service users. Mental Health Practice.

[31] Nash M (2014). Mental health service users' experiences of diabetes care by Mental Health Nurses: an exploratory study. J Psychiatr Ment Health Nurs.

[32] van Nieuwenhuizen A, Henderson C, Kassam A, Graham T, Murray J, Howard LM, Thornicroft G (2012). Emergency department staff views and experiences on diagnostic overshadowing related to people with mental illness. Epidemiol Psychiatr Sci.

[33] Hamilton S, Pinfold V, Cotney J, Couperthwaite L, Matthews J, Barret K, Warren S, Corker E, Rose D, Thornicroft G, Henderson C (2016). Qualitative analysis of mental health service users' reported experiences of discrimination. Acta Psychiatr Scand.

[34] Shefer G, Henderson C, Howard LM, Murray J, Thornicroft G (2014). Diagnostic overshadowing and other challenges involved in the diagnostic process of patients with mental illness who present in emergency departments with physical symptoms--a qualitative study. PLoS One.

[35] Gupta A, Dhami A (2015). Measuring the impact of security, trust and privacy in information sharing: A study on social networking sites. J Direct Data Digit Mark Pract.

[36] (2020). Trusted Research Environments (TRE): A strategy to build public trust and meet changing health data science needs. Draft Green Paper v1.0 for consultation. UK Health Data Research Alliance.

[37] Brett J, Staniszewska S, Mockford C, Herron-Marx S, Hughes J, Tysall C, Suleman R (2014). A systematic review of the impact of patient and public involvement on service users, researchers and communities. Patient.

[38] (2019). Exploring the UK's digital divide. Office for National Statistics.

[39] Mannheim I, Schwartz E, Xi W, Buttigieg SC, McDonnell-Naughton M, Wouters EJ, van Zaalen Y (2019). Inclusion of older adults in the research and design of digital technology. Int J Environ Res Public Health.

[40] Janghorban R, Roudsari R, Taghipour A (2014). Skype interviewing: the new generation of online synchronous interview in qualitative research. Int J Qual Stud Health Well-being.

[41] Mason M (2010). Sample size and saturation in Phd studies using qualitative interviews. Forum Qualitative Social Research/Sozialforschung.

[42] Guest G, Bunce A, Johnson L (2016). How many interviews are enough?. Field Methods.

