# Supplementary figures and images for: Investigating Mental Health Service User Opinions on Clinical Data Sharing: Qualitative Focus Group Study
Source: JMIR Ment Health. 2021 Sep 3;8(9):e30596. doi: 10.2196/30596 (PMC8449295; doi:10.2196/30596)

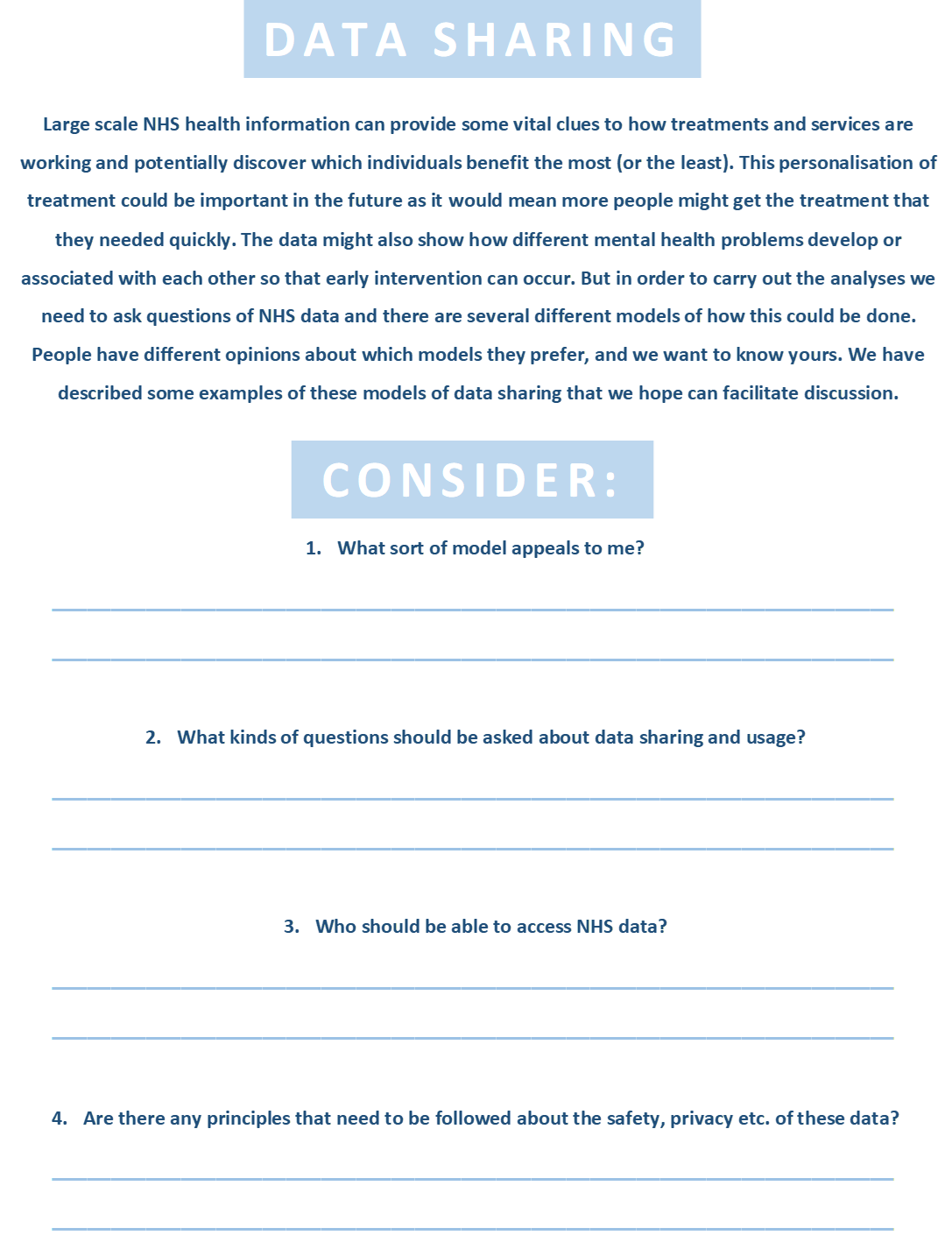


**
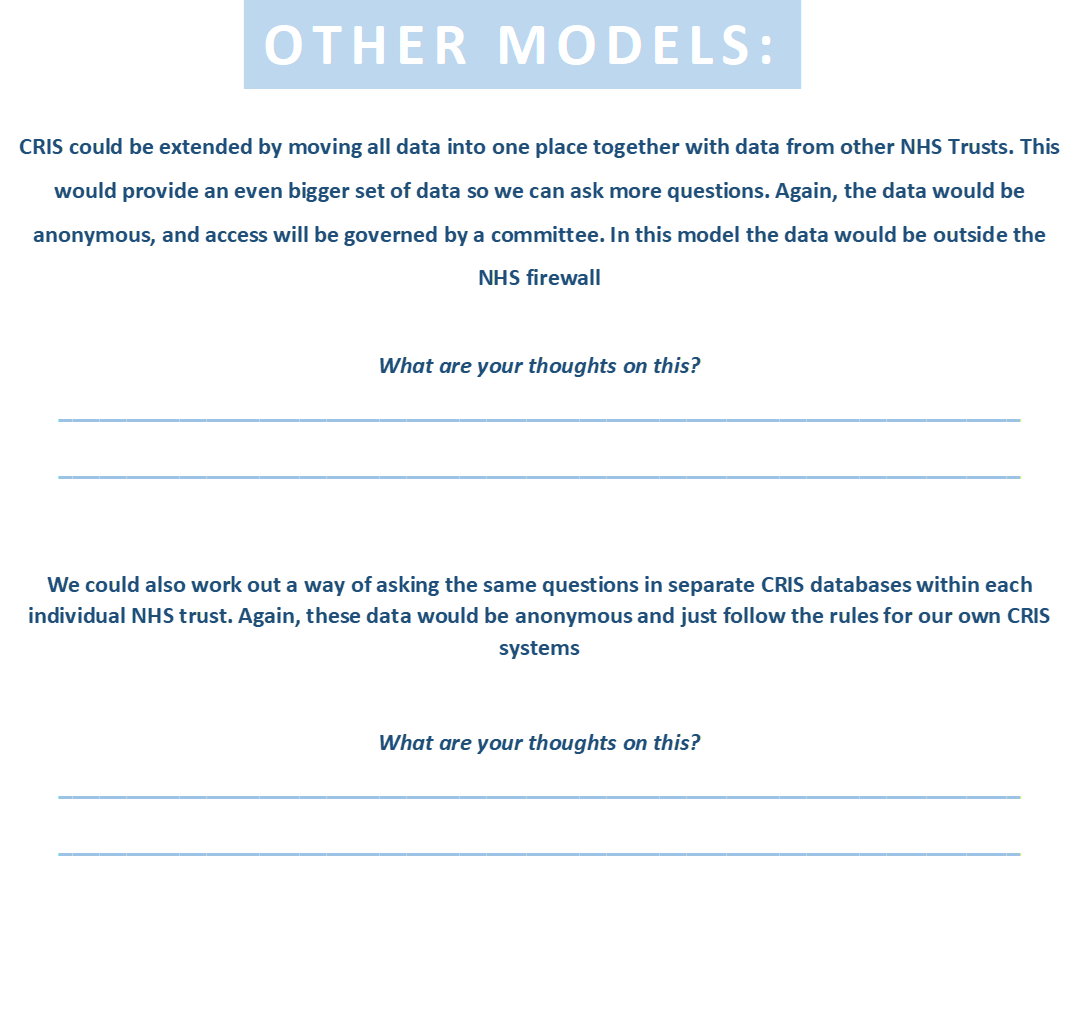
**

Supplement: Multimedia Appendix 1 [file mental_v8i9e30596_app1.docx]
