# Supplementary material for: Investigating Mental Health Service User Opinions on Clinical Data Sharing: Qualitative Focus Group Study
Source: JMIR Ment Health. 2021 Sep 3;8(9):e30596. doi: 10.2196/30596 (PMC8449295; doi:10.2196/30596)
Supplement: Multimedia Appendix 2 [file mental_v8i9e30596_app2.docx]

| **Theme** | **Subtheme** | **Group 1^a^** | **Group 2 ^a^** | **Group 3 ^a^** | **Group 4 ^a^** |
| --- | --- | --- | --- | --- | --- |
| **Purpose** | *For-profit* | ✓ | ✓ | ✓ | ✓ |
|  | *Summary vs raw data* | ✓ | ✓ | ✓ | ✓ |
|  | *Public good* | ✓ | ✓ | ✓ | ✓ |
|  | *Academic research* | ✓ | × | ✓ | ✓ |
|  | *Summary vs raw data* | ✓ | ✓ | ✓ | ✓ |
|  | *Continuity of care* | ✓ | ✓ | ✓ | ✓ |
| **Discrimination** | *Diagnostic overshadowing* | ✓ | ✓ | ✓ | ✓ |
|  | *Mental vs physical health data* | ✓ | ✓ | × | × |
|  | *Institutional discrimination* | ✓ | ✓ | ✓ | ✓ |
| **Accuracy** | *Inaccurate records* | ✓ | ✓ | ✓ | ✓ |
|  | *Transparency* | ✓ | ✓ | ✓ | ✓ |
| **Informed Consent** | *Transparency* | ✓ | ✓ | ✓ | ✓ |
|  | *Awareness* | ✓ | ✓ | ✓ | ✓ |
|  | *Choice* | ✓ | ✓ | ✓ | ✓ |
|  | *Opt-out* | ✓ | ✓ | ✓ | × |
| **Safeguarding** | *Confidentiality* | ✓ | ✓ | ✓ | ✓ |
|  | *Access* | ✓ | ✓ | ✓ | ✓ |
|  | *Anonymity* | ✓ | ✓ | ✓ | ✓ |
|  | *Security* | ✓ | ✓ | ✓ | ✓ |
|  | *Data set size* | ✓ | × | ✓ | ✓ |
|  | *Accountability* | × | ✓ | × | ✓ |
| **Service User Involvement** | *Security* | ✓ | × | ✓ | ✓ |
|  | *Insight* | ✓ | × | ✓ | ✓ |
|  | *Governance* | ✓ | × | ✓ | ✓ |
| **Note**. **^a^** A ‘tick’ indicates whether a theme/sub-theme was discussed in the respective focus group. This was used to calculate data saturation. | | | | |  |
|  | | | | |  |
